# Editorial: From genetic alterations to new molecular targets in pituitary disorders

**DOI:** 10.3389/fendo.2024.1375475

**Published:** 2024-02-07

**Authors:** Jimena Ferraris, Maria Ines Perez-Millan, Juan Pablo Petiti

**Affiliations:** ^1^ Department of Biophysics and Biochemistry, Stockholm University, Stockholm, Sweden; ^2^ Departamento de Fisiología, Biología Molecular y Celular, Facultad de Ciencias Exactas y Naturales, Instituto de Biociencia, Biotecnología y Biología Traslacional (iB3), Universidad de Buenos Aires, Buenos Aires, Argentina; ^3^ Facultad de Ciencias Médicas, Centro de Microscopía Electrónica, Universidad Nacional de Córdoba, Córdoba, Argentina; ^4^ Consejo Nacional de Investigaciones Científicas y Técnicas (CONICET), Instituto de Investigaciones en Ciencias de la Salud (INICSA), Córdoba, Argentina

**Keywords:** pituitary plasticity, pituitary tumors, Wnt/β-catenin pathway, sphingosine-1phosphate, low blood testosterone level

The pituitary gland is a central regulator of growth, reproduction and stress among other physiological functions through the secretion of five different hormone-producing cells types. A main characteristic of the anterior pituitary gland is its plasticity, which allows to adjust to different physiological conditions related to endocrine demands. Hormones are secreted by specific cells in the pituitary gland and this secretion is pulsatile (1). Pulses of pituitary hormones are generated and modified at multiple levels. From a therapeutic point of view, efforts are being focused on the study of normal hypothalamic-pituitary axes function and how dysregulation occurs in a disease context. This is particularly relevant to pituitary tumors, where hormone output is largely independent of hypothalamic stimulation (2).

The present Research Topic “*From genetic alterations to new molecular targets in pituitary disorders*” includes 4 articles to shed light on plasticity of the pituitary gland, cell signaling alterations and the identification of biomarkers in pituitary tumors.

The plasticity is associated with the adjustment and maintenance of anterior pituitary cell number, mediated by specific local growth factors, which may be regulated by stimulatory or inhibitory extra-pituitary factors. In this context, Sosa et al. address the question whether the activation of GPCR-G_αi_ induced by a somatostatin analog (SSTa) can regulate FGF2 proliferative activity in normal pituitary cell populations. In this nicely done study, they showed that FGF2 increased, whereas SSTa decreased lactotroph and somatotroph proliferation. Interestingly, the combined treatment of both factors (FGF2/SSTa) resulted in a decrease in lactotroph and somatotroph cell populations. This response was associated with the arrest of the cell cycle in the G1 stage, a reduction of ERK1/2 and an increase of JNK phosphorylation, demonstrating the involvement of GPCR- Gαi in the modulation of the S-phase entry induced by FGF2 in lactotroph and somatotroph cells. Other signaling pathways involved in tissue homeostasis is the Wnt/β-catenin pathway, whose deregulation often leads to tumor formation (3). In this sense, Wang et al. comprehensively reviewed the alteration of the Wnt/β-catenin pathway in pituitary tumors and the latest developments in inhibitors and pathway-targeted drugs. The Wnt signaling pathway plays a significant part in guiding the development and pathogenesis of pituitary tumors.The review provides an overview of the use of Wnt signaling pathway inhibitors in treating pituitary tumors. Abnormal expression of Wnt-signaling inhibitors may be related to the cell multiplication, invasion, and recurrence. Interestingly they discuss the possibility of combining Wnt pathway inhibitors with immunotherapy to provide a theoretical basis for the combined treatment of pituitary tumors.

In the last years, the availability of new technologies has allowed the generation of molecular data from large cohorts of patients to identify cellular markers and consequently predict the pituitary tumor behavior. With this aim, Sun et al. measured the difference in the concentration of sphingosine-1-phosphate (S1P) in bilateral petrosal sinus blood samples and explored the clinical predictive value of the S1P concentration ratio in determining tumor laterality and postoperative remission in 25 patients with Cushing’s disease (CD). Results indicate that using the interpetrosal S1P or ACTH ratios alone yielded accuracies of 64% and 56% respectively. Notably, the combination of both demonstrated a significantly improved accuracy of 73%. In conclusion, they demonstrate a significant association between the interpetrosal S1P ratio and tumor laterality, as well as in early remission in CD. These findings suggest that the interpetrosal S1P ratio could serve as a useful biomarker in clinical practice. Another interesting point is related with the comorbidities caused by excess levels of circulating hormones. The hypersecretion of GH and IGF-I in acromegaly have deleterious effects on a wide range of tissues and physiologic processes such as hypogonadotropic hypogonadism (HH) (4). Zhang et al. investigated the prevalence of low blood testosterone level (LTL) in a cohort of male acromegaly patients. The 40% of patients were diagnosed as having LTLs and presented significantly higher percentages of macroadenomas, invasion of the cavernous sinus, and compression of the optic chiasm compared to normal testosterone level group. This suggests that male acromegaly patients with a larger and more invasive tumor would also have a greater chance of LTL, which can be explained by the size effect of tumor on the normal function of the pituitary gland by changing pituitary architecture and changing blood flow. In addition, most male patients can recover from LTLs after tumor restriction surgery, and those who recover from acromegaly have a better chance of recovery from LTL.

In summary, the interaction of stimulatory and inhibitory signals in a physiological context, the identification of new therapeutic targets and the management of comorbidities in patients with hypersecreting tumors reflected in the four articles of this Research Topic, represent the three fundamental pillars necessary to understanding of the molecular alteration consequences in the complex pituitary network that may be transferred to clinical practice for a more efficient diagnostic and prognostic of patients with pituitary disorders.

## Author contributions

JF: Writing – review & editing. MP-M: Writing – review & editing. JPP: Writing – original draft, Writing – review & editing.
